# A Systematic Review of Medication Exposure Assessment in Prospective Cohort Studies of Community Dwelling Older Australians

**DOI:** 10.1371/journal.pone.0124247

**Published:** 2015-04-24

**Authors:** Susan G. Poole, J. Simon Bell, Natali Jokanovic, Carl M. Kirkpatrick, Michael J. Dooley

**Affiliations:** 1 Centre for Medicine Use and Safety, Faculty of Pharmacy and Pharmaceutical Sciences, Monash University, Melbourne, Victoria, Australia; 2 Pharmacy Department, Alfred Health, Melbourne, Victoria, Australia; University of Louisville, UNITED STATES

## Abstract

**Introduction:**

It is not known to what extent medication use has been comprehensively assessed in prospective cohort studies of older Australians. Understanding the varying methods to assess medication use is necessary to establish comparability and to understand the opportunities for pharmacoepidemiological analysis. The objective of this review was to compare and contrast how medication-related data have been collected in prospective cohorts of community-dwelling older Australians.

**Methods:**

MEDLINE and EMBASE (1990–2014) were systematically searched to identify prospective cohorts of ≥1000 older participants that commenced recruitment after 1990. The data collection tools used to assess medication use in each cohort were independently examined by two investigators using a structured approach.

**Results:**

Thirteen eligible cohorts were included. Baseline medication use was assessed in participant self-completed surveys (n = 3), by an investigator inspecting medications brought to a clinic interview (n = 7), and by interviewing participants in their home (n = 3). Five cohorts sought participant consent to access administrative claims data. Six cohorts used multiple methods to assess medication use across one or more study waves. All cohorts assessed medication use at baseline and 12 cohorts in follow-up waves. Twelve cohorts recorded prescription medications by trade or generic name; 12 cohorts recorded medication strength; and 9 recorded the daily medication dose in at least one wave of the cohort. Seven cohorts asked participants about their “current” medication use without providing a definition of “current”; and nine cohorts asked participants to report medication use over recall periods ranging from 1-week to 3-months in at least one wave of the cohort. Sixty-five original publications, that reported the prevalence or outcomes of medication use, in the 13 cohorts were identified (median = 3, range 1–21).

**Conclusion:**

There has been considerable variability in the assessment of medication use within and between cohorts. This may limit the comparability of medication data collected in these cohorts.

## Introduction

Medications represent a major health intervention for the prevention and treatment of acute and chronic illness. Between 76% and 95% of community-dwelling older people take one or more prescription medications on a daily basis, and almost half take five or more [1, 2]. While medication use usually leads to improved health outcomes [3], medication-related problems and adverse drug events (ADEs) are leading causes of preventable morbidity and mortality in older people [4].

Prospective cohorts provide an opportunity to assess the patterns and predictors of medication use at a population level. There has been considerable investment in these cohorts of older people in Australia over the past 25 years. These cohorts have been either general population-based or had a specific disease or clinical focus. Medication data have typically been collected for secondary research objectives or to use as covariates in multivariate analyses [5].

Medication use is complex to assess and report. Unlike other clinical parameters there are no universally accepted methods to collect and report medication use in cohort studies. Medication use is highly variable in terms of dose, dose frequency and the specific active pharmaceutical ingredients that are used. Depending on the outcome of interest, both acute and cumulative exposure to medications may be relevant. Data collection methods need to be flexible enough to take into account rapidly evolving therapeutics, yet sufficiently consistent that medication use can be compared over a long-term duration.

Collecting medication data in prospective cohort studies is important because medication use may confound primary outcomes of interest if not appropriately accounted for. For example, cohorts investigating the incidence of falls should assess use of falls-risk medications [6]; cohorts investigating cognitive decline should assess use of medications that impair cognition [7]; and cohorts investigating glycaemic control should assess use of medications that impact blood glucose levels [8].

It is not known to what extent medication use has been comprehensively assessed in prospective cohorts of older Australians. Understanding the varying methods to assess medication use is necessary to establish comparability [9, 10], and to understand the opportunities for pharmacoepidemiological analysis.

The objective of this study was to compare and contrast how medication-related data have been collected in prospective cohorts of community-dwelling older Australians.

## Methods

### Search strategy

A systematic review using MEDLINE and EMBASE was conducted to identify articles describing eligible cohorts. The literature search was limited to articles published between January 1990 and March 2014, inclusive. In MEDLINE, Medical Subject Headings (MeSH) and truncated keywords related to prospective cohorts (“cohort studies” [MeSH] AND “cohort” in title or abstract) AND older people (“aging” [MeSH] OR "aged” [MeSH] OR “aged, 80 and over" [MeSH] OR “geriatrics” [MeSH] OR “old* adj (person* or adult* or people* or patient* or population*)” OR “elder*" OR "geriatric*") were used. All MeSH terms were ‘exploded’ to retrieve results using the selected term and all the more specific terms within the MeSH tree structure. A keyword search via PubMed was performed to identify additional articles not yet indexed into MEDLINE. In EMBASE, the Emtree subject headings “cohort analysis” OR “longitudinal study” OR “prospective study” were used in place of “cohort studies”. To identify Australian publications, (“Australia” [MeSH] OR “Australia*”) were combined with the Boolean operator ‘AND’ in the command-line in each database. The search was limited to Human Studies in the English language.

**Fig 1:**
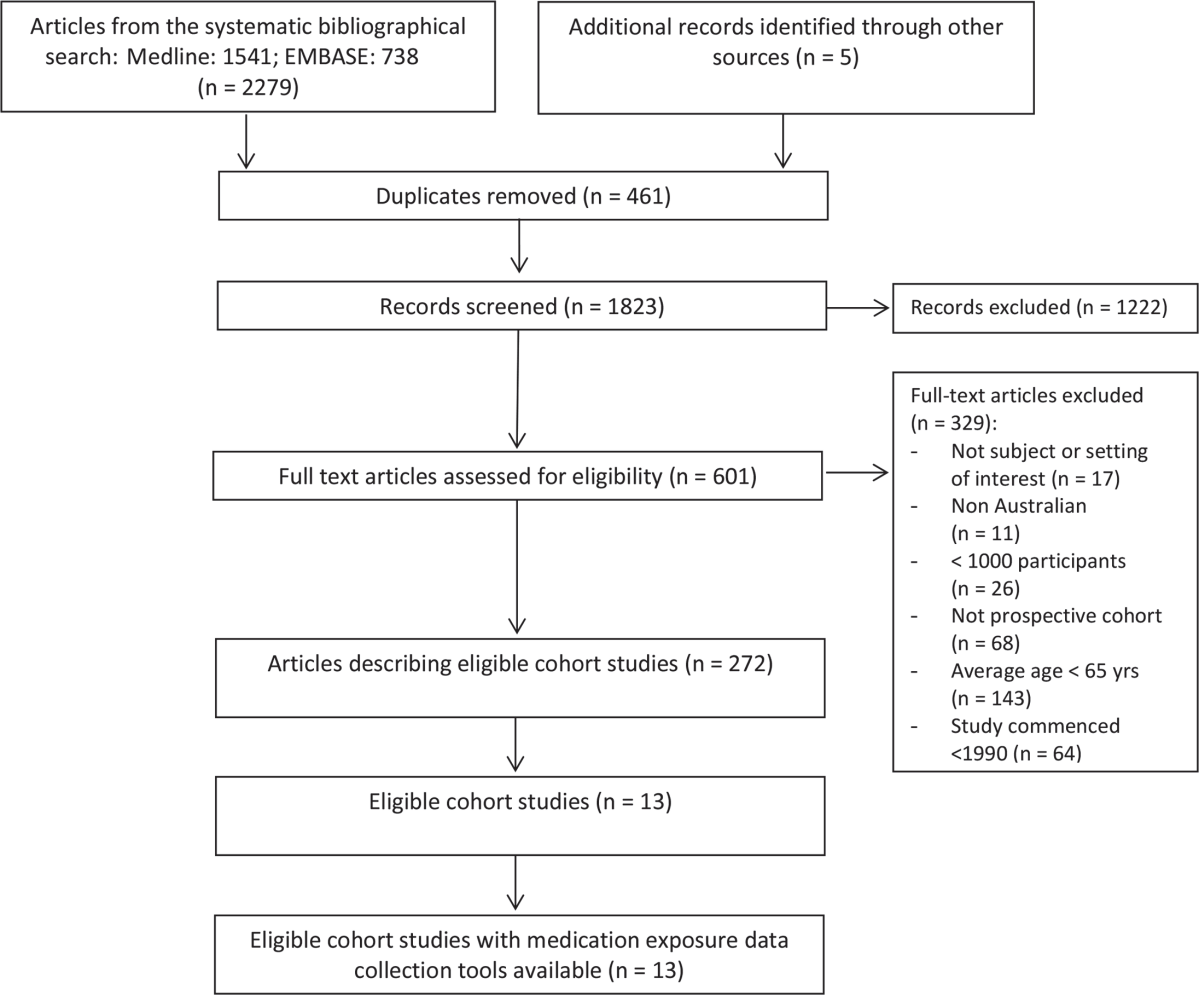
PRISMA flowchart of studies involved in systematic review.

A manual search of Australian Government websites, including The Australian Institute of Health and Welfare (AIHW), the Commonwealth Department of Health, the Australian Bureau of Statistics (ABS) and the Australian Data Archive was conducted to identify relevant publications and reports. Reference lists of all included publications and reports were screened for additional relevant publications. See Fig 1 for PRISMA flowchart of studies involved in systematic review. (The PRISMA Checklist is available as S1 PRISMA Checklist.)

### Inclusion and exclusion criteria

Australian prospective cohorts that commenced baseline participant recruitment from 1990 and included 1000 or more participants were eligible for inclusion. Cohorts with a focus on community dwelling older people (defined as a mean age over 65 years) were considered. Cohorts that included a sub-cohort of community-dwelling older people were also considered. Multiple articles reporting results from the same cohort study were grouped together. If available, the published protocol or cohort profile was reviewed to identify if medication use was listed as an exposure of interest. Only cohort studies for which the full data collection instrument was available in the public domain or by contacting the principal investigators were eligible for inclusion. Retrospective cohorts that utilized administrative claims data as the only source of medication exposure data were excluded. Cohorts that focused on a population of ‘cases’ or participants with a specific disease were excluded.

The review was limited to cohorts of 1000 or more participants because these studies represented the greatest investment of investigator time and resources, were more likely to have the data collection instruments available in the public domain, and were more likely to involve general population-based samples from which results of medication-related analyses could be generalized.

### Data extraction

Data collection tools from the included cohorts were reviewed independently by two investigators using a structured data extraction instrument. The data collection tool from each cohort was reviewed against a list of key aspects of medication exposure assessment (see Table 1).

**Table 1:** Aspects of medication exposure sought in Australian prospective cohort studies.

• Primary and secondary source/s of medication exposure data
• Medications included (i.e. prescription only, over the counter (OTC), complementary and alternative medicines (CAM), treatment for only specifically included conditions or usage of only specifically listed medications)
• Period of time over which participants were asked to report medication use
• Medication strength
• Daily dose and dosing frequency
• Prescribing indication
• Duration of therapy
• Other aspects of medication use, including use of dose administration aids; access to medication advice; adverse drug events
• Number of study waves in which medication exposure was assessed (i.e. whether data were sought in the longitudinal phases of the study)
• Participant consent to link self-reported data to administrative claims data

The chief investigator of each of the included cohorts was given the opportunity to verify interpretation of the data extraction.

### Medication-related publications from the included cohorts

To identify whether pharmacoepidemiological analyses had been published from each cohort, publication lists from the study websites were examined. An author search of MEDLINE, EMBASE and PubMed was also conducted using the names of the principal investigators of each cohort. All publications identified were screened by title and abstract to identify publications with a quantitative pharmacoepidemiological analysis. The full text versions of all medication-related publications were reviewed to assess whether the methods utilized cross-sectional or longitudinal medication exposure and outcome data. A description of the coding system employed to categorize medications was extracted.

### Analysis

The medication exposure assessment in each cohort was summarized and compared descriptively. The medication-related publications arising from each cohort were reviewed and grouped according to the type of analyses conducted (i.e. cross-sectional baseline medication exposure and association with follow-up outcomes, prevalence of medication use at multiple waves).

## Results

Thirteen cohorts involving baseline participant recruitment from 1990 to 2006 were included (see Table 2). All 13 cohorts had a health focus and 11 cohorts included a medical examination of participants in the longitudinal waves of the study. Four cohorts had a broad focus, examining ‘healthy aging’ or the burden of disease in an ageing population [11–14]; six focused on the development of a specific disease or range of related diseases or health issues as the cohort ages [15–20]; three had a focus on gender-related health issues [21–23], and two are large cohorts based on questionnaire-derived responses and data-linkage [11, 21]. The included cohorts had a median of four ‘waves’ of longitudinal data collection (range 2–11); with seven cohorts ongoing and six complete.

**Table 2:** Australian prospective cohort studies of community dwelling older people (n = 13).

Study Name (Abbreviation) Study period (waves completed to date, waves with medication use assessed)	Primary research question (methodology)	Recruitment method (#) Sample size at baseline; Percent of older adults	Primary medication exposure question/s (recall period in bold)
45 and Up (45 & Up) [11] 2006-ongoing (2,2)	Broad determinants of health (Survey)	Random sample (M) 267,153; 40% ≥ 65 yrs	All waves: Have you taken any medications, vitamins or supplements for most of the last **4 weeks**?
Australian Imaging, Biomarkers and Lifestyle (AIBL) [15] 2006-ongoing (5, 5)	Factors influencing development of Alzheimer's Disease (Clinical assessment & survey)	Media appeal and physician recruitment 1,112; 84% ≥ 65 yrs	All waves: Do you **currently** take any medications (including non-prescription; i.e. herbal supplements, vitamins etc)?
Australian Longitudinal Study of Ageing (ALSA) [12] 1992–2010 (11, 6)	Factors associated with age-related changes in health (Clinical assessment & survey)	Random sample (E) + spouse of primary respondents (aged ≥ 65 yrs) & other household members ≥ 70 yrs invited 2,087; 100% ≥ 70 yrs	Wave 1: … any medicines prescribed by a doctor that you have taken or were supposed to take in the last **two weeks**…. all other medicines not prescribed by a doctor such as aspirin, headache pills, laxatives, cough and cold medicines, vitamins, minerals and dietary supplements. (Do not include ointments.); Wave 3: Are you still taking *[medications identified in Wave 1*]?. … new medicines you have started taking in the last **two years**. These include… [as per Wave 1]; Wave 6, 7, 9: as per Wave 1; Wave 11: as per Wave 1 or consent for administrative claims data
Australian Longitudinal Study of Women’s Health (ALSWH) [21] * 1996-ongoing (7, 7)	Influences of health among women (Survey)	Random sample (M) 12,400; 100% ≥ 70 yrs	Wave 1–3: During the past **4 weeks**, how many different types of medication (eg tablets/medicine) have you used which were prescribed by a doctor; Wave 1–2: During the past **4 weeks**, how many different types of medication (eg tablets/medicine) have you used which were bought without a prescription at the chemist, supermarket or health food shop; Wave 4: Write down the names of all you medications prescribed by a doctor (no recall period specified); Wave 5–7: Consent for administrative claims data
Australian Diabetes, Obesity and Lifestyle Study (AusDIAB) [16] 1999–2012 (3, 3)	Natural history of diabetes (Clinical assessment & survey)	Random sample (ABS CDs) 11,200; 21% ≥ 65 yrs (subset analyses of 65–74 & ≥ 75 yrs)	Wave 1: Are you **currently** taking tablets for high blood pressure? Are you **currently** taking tablets to lower your cholesterol/triglycerides?; Wave 2–3: … all prescription medications used in the **last three months** for a period of at least 2 weeks
The Blue Mountains Eye Study (BMES) [17] 1992–2007 (4, 4)	Visual impairment & eye diseases (Clinical assessment & survey)	Door-to-door census: all dwellings in selected area 3,654; 53% ≥ 65 yrs	Wave 1: … tablets or vitamins you’ve taken in the **last week**.; Wave 2: (data collection tool unavailable); Wave 3: … the tablets, vitamins or other medications you are **currently** taking.; Wave 4: (data collection tool unavailable)
Canberra Longitudinal Study of Ageing (CLS) [18] 1990–2002 (4, 4)	Depression & cognitive impairment (Clinical assessment & interview)	Random sample (E) + sample from nursing homes & hostels for the elderly 1,100; 100% ≥ 70 yrs	All waves: Are you **currently** taking any tablets for high blood pressure?; Wave 2: Was the **ever** a **long period** when you took… aspirin daily of almost daily? … non-aspirin pain relievers daily of almost daily? … non-steroidal anti-inflammatory compounds daily of almost daily?; Wave 3 & 4: Was the **ever** a period of **a year or more** when you took… aspirin daily of almost daily? Has this been in the last 4 years? … non-aspirin pain relievers daily of almost daily? Has this been in the last 4 years? … non-steroidal anti-inflammatory compounds daily of almost daily? Has this been in the last 4 years?
Concord Health and Ageing in Men Project (CHAMP) [22] 2005-ongoing (2, 2)	Men’s health & ageing (Clinical assessment & survey)	Random sample (E) 1,700; 100% ≥ 70 yrs	All waves: …any medication, (taken) daily or almost daily, for at least the **past month**? This includes both prescription and non-prescription medication.
Health In Men Study (HIMS) [23] 1996-ongoing (5, 5)	Men’s health & ageing (Clinical assessment & survey)	Random sample (E) 12,203; 100% ≥ 65 yrs	Wave 1: In the **last month** have you been… taking tablets for blood pressure?. . . taking tablets or other treatment for angina? …on aspirin tablets to prevent or treat heart disease? … taking medication prescribed by a doctor to lower your blood cholesterol?; Wave 2: … all the tablets, inhalers, and other medicines you are **currently** taking… include any non-prescription medicine you may have obtained from a chemist or supermarket; Wave 3: … all of your **current** medicines prescribed by a doctor… include tablets, capsules, aspirin, liquids or syrups or mixtures, puffers, sprays, nebulisers, creams, ointments, pastes, patches, suppositories, injections, etc taken now.; Wave 4&5: … all of your **current** medicines prescribed by a doctor. . . include tablets, capsules, aspirin, liquids /syrups /mixtures, puffers, sprays, nebulisers, creams, ointments, pastes, patches, suppositories, injections, etc taken now. AND … all of your **current** medicines, vitamins etc bought from a pharmacy (chemist) without a prescription. . . include tablets, capsules, aspirin, liquids /syrups /mixtures, puffers, sprays, nebulisers, creams, ointments, pastes, patches, suppositories, etc taken now.
Hunter Community Study (HCS) [13] 2004-ongoing (3, 3)	Assess factors important in the health, well-being, social functioning & economic consequences of ageing (Clinical assessment & survey)	Random sample (E) 3,253; 55% ≥ 65 yrs	All waves: … ALL your **current** medications (including tablets, medicines, painkillers, antacids…. ALL the current vitamins/ minerals/ herb preparations that you take.
Melbourne Longitudinal Studies on Healthy Ageing (MELSHA) [14] 1994–2005 (11, 1)	Biomedical, psychological, behavioural & social influences on ageing (Clinical assessment & survey)	Random sample (E) 1,000; 100% ≥ 65 yrs	Wave 1: … any medicines prescribed by a doctor that you have taken or were supposed to take in the last **two weeks**… anything else that you have taken to relieve pain, to help you sleep or to relax in the last two weeks?
South Australian Dental Longitudinal Study (SADLS) [19]1991–2002 (4, 3)	Assess oral health status of older people (Clinical assessment & survey)	Random sample (E) 1,650; 75% ≥ 65 yrs	All waves: … any prescribed medicines or medicines bought over the counter… that you have taken or were supposed to take regularly in the past **2 weeks**
Sydney Memory and Ageing Study (MAS) [20] 2005-ongoing (4, 4)	Examine characteristics & prevalence of MCI** & determine change in cognitive function over time (Clinical assessment & survey)	Random sample (E) 1,037; 100% ≥ 70 yrs	All waves: … all of your **current** medications, including over-the-counter medications e.g. vitamins and herbal/natural therapy

* ALSWH: surveys relating to “Older cohort” (DOB: 1921–1926) only

** MCI: Mild Cognitive Impairment

#: M: Medicare Australia enrolment database, E: Electoral Role, ABS CDs: Australian Bureau of Statistics Collector Districts

### Assessment of medication use

Table 3 summarizes the methods used to obtain medication exposure. All cohort studies utilized participant self-report to assess medication use at baseline: three cohorts used participant self-completed surveys [11, 13, 21]; seven cohorts asked participants to bring all their medications to a clinic interview [15–17, 19, 20, 22, 23], and participants were interviewed in their homes in three cohorts [12, 14, 18]. In addition, three cohorts obtained participant consent to conduct data-linkage with the administrative claims data (i.e. Pharmaceutical Benefits Scheme (PBS) and Repatriation Pharmaceutical Benefits Scheme (RPBS)) at baseline [11, 13, 20].

**Table 3:** Source of medication exposure assessment.

Source	Studies utilising source at baseline (n)	Studies utilising source in longitudinal ‘waves’ (n)
Self-report: Survey	3 [11, 13, 21]	4 [11, 13, 21, 23]
Self-report by interview: Clinic visit	7 [15, 16, 17, 19, 20, 22, 23]	7 [15, 16, 17, 19, 20, 22, 23]
Self-report by interview: Home visit	3 [12, 14, 18]	2 [12, 18]
PBS/RPBS consent *	3 [11, 13]	5 [11, 12, 13, 17, 21]
Pharmacy generated list	-	3 [16, 17, 21]
Multiple sources	3 [11, 13, 20]	6 [11, 12, 13, 17, 21, 23]

* Consent provided for linkage to PBS/RPBS: Pharmaceutical Benefits Scheme/ Repatriation Pharmaceutical Benefits Scheme (pharmaceutical administrative claims data)

In regards to follow-up data collection, only one cohort did not assess medication use after baseline [14]. Six cohorts used the same source or sources of data and data collection methods consistently across all study waves that included an assessment of medication [11, 13, 15, 19, 20, 22], the remaining six cohorts modified the medication-related questions at different time points of the study [12, 16, 17, 18, 21, 23] and two of these studies changed the source of medication data [12, 21]. Three cohorts invited participants to provide a current medication list from their regular pharmacy as an additional source of medication data [16, 17, 21].

Reporting of medication exposure time was inconsistent between and within studies. Five of the 13 cohorts used different exposure time windows at different time points [16, 17, 18, 21, 23] and one cohort did not define the exposure time window in one wave [21]. Seven cohorts requested the participant to report ‘current’ medications during at least one wave [13, 15–18, 20, 23], see Table 2.

Table 4 summarizes the information regarding the comprehensiveness of the assessments. Past medication use was sought in nine cohorts in at least one wave. Eight cohorts requested information about ‘ever’ use of specified medications [11, 16–18, 20–23], one asked for ‘ever use’ of past medications taken for more than three months [17], and one confirmed persistent use of baseline medication at wave 2 [12].

**Table 4:** Comprehensiveness of medication exposure assessment.

	Prescription medication name	OTC name	Exposure time defined	Medication strength	Dose/frequency	Participant reported indication	Participant reported duration of therapy	Past medication use	Consent for PBS/RPBS linkage	Questions consistent longitudinally
45 & Up [11]	B, L	B, L	B, L	B**				B^#^	B, L	Y
AIBL [15]	B,L	B, L	B*, L*		B, L	B, L	B, L			Y
ALSA [12]	B, L	B, L	B, L	B, L**	B	B, L	B, L	L^###^	L	
ALSWH [21]	L		B, L	L**				B^#^	L	
AusDiab [16]	L		B*, L	L	L			B^#^		
BMES [17]	B, L	B, L	B, L*	L	L		B, L	B^#^ ^,^ ^##^, L^#^ ^,^ ^##^	L	
CLS [18]			B*, L*					L^#^		
CHAMP [22]	B, L	B, L	B, L	B, L	B, L		B, L	B^#^, L^#^		Y
HIMS [23]	L	L	B, L*	L			L	L^#^		
HCS [13]	B, L	B, L	B*, L*	B, L	B, L				B, L	Y
MELSHA [14]	B		B	B***	B	B				
SADLS [19]	B, L	B, L	B, L	B, L	B, L					Y
Syd-MAS [20]	B, L	B, L	B*, L*		B, L		B, L	B^#^, L^#^	B	Y

B: Baseline

L: Longitudinal waves

Y: Medication questions consistent during all waves of study

OTC: ‘Over the Counter’ medication (ie non-prescription medications, including vitamins, herbal supplements etc)

PBS/RPBS: Pharmaceutical Benefits Scheme/ Repatriation Pharmaceutical Benefits Scheme (pharmaceutical administrative claims data)

* Exposure time = “current”

** strength of prescription medications via PBS data

*** for analgesic or psychotropic medicines only (defined list)

# “Ever” use of a small number of named medications of interest

## “Any other tablets taken for more than 3 months in the past”

### Wave 2: confirm persistent use of medications identified at baseline

Twelve cohorts requested the name of all prescription medications being used in at least one wave of the study and nine cohorts requested the name of current over-the-counter (OTC) and/or vitamins, minerals and herbal supplements.

Medication strength and dose/frequency information was assessed at baseline and at least one follow-up wave in only three cohorts [13, 19, 22], and one cohort recorded neither strength nor dose/frequency information [18]. The remaining cohorts collected either strength or dose/frequency data in at least one wave.

Other aspects of medication use assessed included the use of dose administration aids or whether the participant had difficulty managing their medications (n = 3) [14, 21, 23]; whether they had sought health advice from a pharmacist (n = 4) [12, 13, 16, 18]; whether their regular doctor was aware they were taking the medication (n = 2) [12, 14] and whether they had experienced any medication-related adverse events (n = 1) [14].

### Medication-related publications from the cohorts

S1 Table (in Supporting Information) provides information and a reference list of medication-related publications arising from the included cohorts. Sixty-five publications, from the 13 included cohorts, had a specific medication focus (median = 3, range 1–21). In total, 27 (42%) were cross-sectional and reported the prevalence and factors associated with baseline medication use, 19 (29%) reported the association between the prevalence of baseline medication use and the incidence of one or more outcomes, and 17 (26%) reported the prevalence of medication use at multiple waves. One cohort also described a protocol for applying pharmacoepidemiological analyses and another described the development of a unique coding system for medications to facilitate the pharmacoepidemiological analysis. An additional 22 publications from five of the included cohorts included the assessment of vitamin and minerals; herbal supplements or complementary and alternative medicine (CAM) usage in the cross-sectional (n = 15) or longitudinal (n = 7) analysis of health outcomes.

Eight of the cohorts described the coding system used to classify medications for pharmacoepidemiological analysis in at least one of the publications reviewed. Four cohorts utilized the WHO Anatomical Therapeutic Chemical (ATC) Classification System; two cohorts grouped medications by ‘drug class’, utilising the published product information; one utilized the MedCap classification system [24]; and one utilized Iowa Drug Information System (IDIS) drug code numbers. Publications from five cohorts did not describe the coding of medications.

## Discussion

The main finding of this review was the variability, within and between cohort studies, in the methods utilized to assess medication use. This has resulted in inconsistencies in the comprehensiveness of the data collected. The possible impact of these inconsistencies deserves further investigation.

Variability and inconsistency in the methods to assess medication use within a study may limit the opportunity to conduct longitudinal analyses. The five most recent cohorts used a consistent approach to assess mediation use within the study [11, 13, 15, 20, 22]. This may reflect a maturing in the understanding of the importance of medications to health outcomes. However, the benefits of using the same questions about medication exposure at each study wave may need to be traded-off against questionnaire length and ease of administration.

All cohorts used participant self-report as the primary source of medication data. Asking participants to self-report their list of medications has the advantage that only medications actually used by the participants are captured. However, participant self-report is subject to recall error and potential unwillingness to report [25]. Six cohorts obtained participant consent to access administrative claims data. Administrative claims data can be used to supplement self-report data and have been described as the ‘gold-standard’ for medication exposure [26]. However, administrative claims data usually only include records of reimbursed medications, do not include non-prescription medications or CAMs, and do not consider non-adherence [27, 28]. Other investigators have considered participant home interview as the gold standard for assessing medication use [29]. This method allows the investigator to inspect the contents of the medication cabinet. However, conducting home interviews is very resource intensive. It is not clear from the published literature whether any of the 13 cohorts used medication lists obtained from each participant’s general medical practitioner or community pharmacy to supplement or validate participant self-reported medication lists. Three cohorts passively requested these data [16, 17, 21], however it is not clear to what extent these data were obtained. In those studies which conducted participant interviews, it is not clear whether all interviews were performed by the same researcher. It is unclear to what extent the clinical background of the researcher is important for collecting a complete medication list in prospective cohort studies.

It is desirable to assess the prevalence, dose, duration and change in medication use over the course of a study [30]. Each of the 13 cohort studies assessed the prevalence of medication use at baseline. Incident user study designs are considered superior to prevalent user study designs for investigating medication outcomes. This is because prevalent users are the ‘survivors’ of the early period of medication use and, therefore, may be less susceptible to adverse events [31]. In addition, covariates of medication use may be impacted by medication use itself and adjustment for these covariates on the causal pathway between the medication exposure and outcome may result in bias [31]. Only two cohorts obtained ongoing participant consent, from baseline, for linkage to administrative claims data that would enable the capture of incident medication use [11, 13].

Unless medication use is assessed at multiple time-points it is not possible to analyse medication use as a time-dependent exposure. Misclassification of exposure, by incorrectly identifying the use, dose or duration of medication use may result in biased effect estimates [32]. One of the 13 cohorts assessed medication exposure at baseline only [14]. Six of the included cohorts sought information about the ‘duration of treatment’ for prevalent medication use at baseline or in a later wave of the study. Establishing the treatment duration may be important for establishing cumulative exposure.

There was considerable variability in time periods over which participants were asked to report medication use [33]. Seven cohorts asked the participant to consider medications they take ‘currently’ without providing an operational definition of current. Other cohorts used an exposure time window ranging from 1 week to 3-months. A shorter window is likely to improve the reliability and validity of the participant report; however it may result in medications used infrequently being missed [34].

Only one of the included cohorts sought data about *any* medications that had been used previously [17]. Eight of the cohorts did seek information about ‘ever’ use of specific medications, providing the researchers the opportunity of assessing associations between health outcomes and exposure to medications that the participant was not taking at the time of report [11, 16–18, 20–23].

One aspect of medication exposure that has remained largely unexplored in Australian cohorts is that of adherence. Adherence to a prescribed medication is an integral component of medication effectiveness [35]. It is possible to estimate medication persistence from administrative claims data [36]. However, use of claims data alone does not permit exploration of consumer perceptions, preferences, attitudes and opinions about health and medication use [37–39]. Three of the included cohorts did seek information about the participant’s ability to manage medications or use of dosage administration aids [14, 21, 23], both of which may influence the ability of the consumer to adhere to the prescribed medication regimen [40].

At least one medication-related paper has been published from each of the 13 included cohorts. Forty two percent of the publications (n = 27) reported cross-sectional analysis; 19 (29%) reported the association between the prevalence of baseline medication use and the incidence of one or more health outcomes, and 17 (26%) reported the prevalence of medication use at multiple waves. The majority (70%) of the medication-related publications from the included cohorts have reported the prevalence of medication use at baseline without taking advantage of the longitudinal assessments of medication use [32].

There are European initiatives to ensure consistency of pharmacoepidemiological methods [41]. Similarly, establishing consistency in the assessment of medication exposure will enable greater comparison within and between cohort studies. This review has identified aspects of medication exposure that would be worthwhile to consider when establishing a standard approach to the design and analysis of cohort studies.

### Strengths and limitations

One strength of this study is that the investigators of each of the included cohorts were given the opportunity to verify interpretation of the data extraction. We reviewed the data collection methods and tools for each phase of the included cohorts. It was not possible to confirm the quality of data actually collected in each cohort or how missing data were handled in the analyses. The review inclusion criteria resulted in number of well-known Australian cohorts being excluded. A number of the excluded cohorts included medication exposure as a covariate of interest. However, appraisal of these cohorts demonstrated similar issues as identified in this review [5, 42–44]. Not all study websites contained a current comprehensive list of publications arising from the study. It is possible that reports of some pharmacoepidemiological analyses were not retrieved.

### Future directions

Based on the findings of this review we recommend that people with expertise in pharmacoepidemiology are involved in the initial design of new cohort studies. There are also opportunities for further research arising from this review. There is a need to develop consensus around acceptable methods to collect and report medication use in cohort studies. The convergence towards consistency in methods that has been identified, in later cohorts, in this study, may serve as basis for such consensus. Recommendations for standardized definitions and a minimum data set for the assessment of medication exposure would reduce future variability and improve reliability of future cohorts. If a standardized tool was developed to assess medication use it would be of potential benefit in other branches of clinical research, including clinical trials and cross-sectional surveys [2, 45].

## Conclusion

There has been variability within and between cohorts in the methods used to assess medication exposure. Opportunities exist to improve the methods utilized through the development of standardized definitions and a minimum data set for the assessment of medication exposure within cohorts. This may reduce future variability and improve comparability of results of future cohort studies.

## Supporting Information

S1 PRISMA Checklist (PDF)

S1 Table Medication-related publications arising from included cohort studies. (PDF)
