# Supplementary material for: A Systematic Review of Medication Exposure Assessment in Prospective Cohort Studies of Community Dwelling Older Australians
Source: PLoS One. 2015 Apr 24;10(4):e0124247. doi: 10.1371/journal.pone.0124247 (PMC4409061; doi:10.1371/journal.pone.0124247)
Supplement: S1 Table — (PDF) [file pone.0124247.s002.pdf]

Table S1: Medication-related publications arising from included cohort studies

Cohort study	Medication-focussed publications								CAM*-focussed publications	
	Cross-sectional (prevalence of factors associated with baseline medication use)		Prevalence of baseline medication use and incidence of outcomes		Prevalence of medication use at multiple waves		Other			
	Number of publications (27)	Reference	Number of publications (19)	Reference	Number of publications (17)	Reference	Number of publications (2)	Reference	Number of publications (22)	Reference
45 & Up [1]	2	[2,3]	0		2	[4,5]	1	[6]	3	[7-9]
AIBL [10]	1	[11]	0		0		0		0	
ALSA [12]	0		1	[13]	2	[14,15]	1	[16]	1	[17]
ALSWH [18]	4	[19-22]	0		5	[23-27]	0		16#	[28-43]
AusDiab [44]	3	[45-47]	0		0		0		0	
BMES [48]	8	[49-56]	9	[57-65]	4	[66-69]	0		2	[70,71]
CLS [72]	0		0		1	[73]	0		0	
CHAMP [74]	6	[75-80]	4	[81-84]	0		0		0	
HIMS [85]	1	[86]	1	[87]	0		0		1#	[29]
HCS [88]	1	[89]	0		0		0		0	
MELSHA [90]	1	[91]	2	[92,93]	0		0		0	
SADLS [94]	0		0		3	[95-97]	0		0	
Syd-MAS [98]	0		2	[99,100]	0		0			

* CAM: Complementary and alternative medicines

1 publication [29] includes data arising from ALSWH [18] and HIMS [85]

References

1. 45 and Up Study Collaborators, Banks E, Redman S, Jorm L, Armstrong B, et al. (2008) Cohort profile: the 45 and up study. *Int J Epidemiol* 37: 941-947.
2. Kemp A, Preen DB, Saunders C, Boyle F, Bulsara M, et al. (2014) Women commencing anastrozole, letrozole or tamoxifen for early breast cancer: the impact of comorbidity and demographics on initial choice. *PLoS One* 9(1): e84835. doi:10.1371/journal.pone.0084835.
3. Chiu CL, Lujic S, Thornton C, O'Loughlin A, Makris A, et al. (2012) Menopausal hormone therapy is associated with having high blood pressure in postmenopausal women: observational cohort study. *PLoS One* 7(7): e40260. doi:10.1371/journal.pone.0040260.
4. Rogers KD, Kemp A, McLachlan AJ, Blyth F (2013) Adverse Selection? A Multi-Dimensional Profile of People Dispensed Opioid Analgesics for Persistent Non-Cancer Pain. *PLoS ONE* 8(12): e80095. doi:10.1371/journal.pone.0080095.
5. Warren JR, Falster MO, Fox D, Jorm L (2013) Factors influencing adherence in long-term use of statins. *Pharmacoepidemiol Drug Saf* 22: 1298-1307.

6. Gnjidic D, Le Couteur DG, Pearson SA, McLachlan AJ, Viney R, et al. (2013) High risk prescribing in older adults: prevalence, clinical and economic implications and potential for intervention at the population level. *BMC Public Health* 13: 115.

7. Adams J, Sibbritt D, Lui C-W, Broom A, Wardle J (2013) Ω-3 fatty acid supplement use in the 45 and Up Study Cohort. *BMJ Open* 3: e002292. doi:10.1136/ bmjopen-2012-002292.

8. Rahman SZ, Basilakis J, Rahmadi A, Lujic S, Musgrave I, et al. (2013) Use of serotonergic antidepressants and St John's wort in older Australians: a population-based cohort study. *Australas Psychiatry* 21: 262-266.

9. Sibbritt D, Adams J, Lui CW, Broom A, Wardle J (2012) Who uses glucosamine and why? A study of 266,848 Australians aged 45 years and older. *PLoS One* 7(7): e41540. doi:10.1371/journal.pone.0041540.

10. Ellis KA, Bush AI, Darby D, De Fazio D, Foster J, et al. (2009) The Australian Imaging, Biomarkers and Lifestyle (AIBL) study of aging: methodology and baseline characteristics of 1112 individuals recruited for a longitudinal study of Alzheimer's disease. *Int Psychogeriatr* 21: 672-687.

11. Sittironnarit G, Ames D, Bush AI, Faux N, Flicker L, et al. (2011) Effects of anticholinergic drugs on cognitive function in older Australians: results from the AIBL study. *Dement Geriatr Cogn Disord* 31: 173-178.

12. Andrews G, Clark M, Luszcz M (2002) Successful Aging in the Australian Longitudinal Study of Aging: Applying the MacArthur Model Cross–Nationally. *J Soc Issues* 58: 749-765.

13. Vitry A, Wong SA, Roughead EE, Ramsay E, Barratt J (2009) Validity of medication-based co-morbidity indices in the Australian elderly population. *Aust N Z J Public Health* 33: 126-130.
14. Vitry AI, Hoile AP, Gilbert AL, Esterman A, Luszcz MA (2010) The risk of falls and fractures associated with persistent use of psychotropic medications in elderly people. *Arch Gerontol Geriatr* 50: e1-4.
15. Zhang Y, Chow V, Vitry AI, Ryan P, Roughead EE, et al. (2010) Antidepressant use and depressive symptomatology among older people from the Australian Longitudinal Study of Ageing. *Int Psychogeriatr* 22: 437-444.
16. Thomson WM (1997) A medication capture and analysis system for use in epidemiology. *Drugs Aging* 10: 290-298.
17. Goh LY, Vitry AI, Semple SJ, Esterman A, Luszcz MA (2009) Self-medication with over-the-counter drugs and complementary medications in South Australia's elderly population. *BMC Complement Altern Med* 9: 42.
18. Lee C, Dobson AJ, Brown WJ, Bryson L, Byles J, et al. (2005) Cohort Profile: the Australian Longitudinal Study on Women's Health. *Int J Epidemiol* 34: 987-991.
19. Hasan S, Byles JE, Mishra G, Harris MA (2001) Use of Sleeping Medication and Quality of Life among Older Women who Report Sleeping Difficulty. *Australas J Ageing* 20: 29-35.
20. Lowe J, Young AF, Dolja-Gore X, Byles J (2008) Cost of medications for older women. *Aust N Z J Public Health* 32: 89.

21. Tooth LR, Hockey R, Treloar S, McClintock C, Dobson A (2012) Does government subsidy for costs of medical and pharmaceutical services result in higher service utilization by older widowed women in Australia? *BMC Health Serv Res* 12: 179.

22. Dolja-Gore X, Pit SW, Parkinson L, Young A, Byles J (2013) Accuracy of self-reported medicines use compared to pharmaceutical claims data amongst a national sample of older Australian women. *Open J Epidemiol* 3: 25-32.

23. Duke JM, Sibbritt DW, Young AF (2007) Is there an association between the use of oral contraception and depressive symptoms in young Australian women? *Contraception* 75: 27-31.

24. Stewart Williams JA, Wallick CJ, Byles JE, Doran CM (2013) Assessing patterns of use of cardio-protective polypill component medicines in Australian women. *Drugs Aging* 30: 193-203.

25. Lucke JC, Herbert D, Watson M, Dobson A (2011) Contraceptive Changes after Reproductive Events among Australian Women Born in 1973 to 1978: A Longitudinal Study from 1996 to 2009. *Womens Health Issues* 21: 438-443.

26. Lucke JC, Watson M, Herbert D (2009) Changing patterns of contraceptive use in Australian women. Contraception 80: 533-539.

27. Berecki-Gisolf J, Hockey R, Dobson A (2008) Adherence to bisphosphonate treatment by elderly women. Menopause 15: 984-990.

28. Adams J, Sibbritt D, Lui CW (2011) The use of complementary and alternative medicine during pregnancy: a longitudinal study of Australian women. *Birth* 38: 200-206.

29. McLaughlin D, Adams J, Sibbritt D, Lui C-W (2012) Sex differences in the use of complementary and alternative medicine in older men and women. *Australas J Ageing* 31: 78-82.

30. Meurk C, Broom A, Adams J, Sibbritt D (2013) Rurality, mobility, identity: Women's use of complementary and alternative medicine in rural Australia. *Health Place* 20: 75-80.

31. Murthy V, Sibbritt D, Adams J, Broom A, Kirby E, et al. (2014) Self-prescribed complementary and alternative medicine use for back pain amongst a range of care options: Results from a nationally representative sample of 1310 women aged 60–65 years. *Complement Ther Med* 22: 133-140.

32. Adams J, Sibbritt D, Lui CW (2011) The urban-rural divide in complementary and alternative medicine use: a longitudinal study of 10,638 women. *BMC Complement Altern Med* 11: 2.

33. Sibbritt D, Adams J, Murthy V (2013) The prevalence and determinants of Chinese medicine use by Australian women: analysis of a cohort of 10,287 women aged 56-61 years. *Am J Chin Med* 41: 281-291.

34. Sibbritt D, Adams J, Easthope G, Young A (2003) Complementary and alternative medicine (CAM) use among elderly Australian women who have cancer. *Supp Care Cancer* 11: 548-550.

35. Adams J, Sibbritt D, Broom A, Loxton D, Pirotta M, et al. (2011) A comparison of complementary and alternative medicine users and use across geographical areas: a national survey of 1,427 women. *BMC Complement Altern Med* 11: 85.

36. Sibbritt DW, Adams J, Young AF (2004) A longitudinal analysis of mid-age women's use of complementary and alternative medicine (CAM) in Australia, 1996-1998. *Women Health* 40: 41-56.

37. Steel A, Adams J, Sibbritt D, Broom A, Frawley J, et al. (2013) The Influence of Complementary and Alternative Medicine Use in Pregnancy on Labor Pain Management Choices: Results from a Nationally Representative Sample of 1,835 Women. *J Altern Complement Med* 20: 87-97.

38. Poulsen EE, Sibbritt D, McLaughlin D, Adams J, Pachana NA (2013) Predictors of Complementary and Alternative Medicine (CAM) use in two cohorts of Australian women. *Int Psychogeriatr* 25: 168-170.

39. Sibbritt DW, Adams J (2010) Back pain amongst 8,910 young Australian women: a longitudinal analysis of the use of conventional providers, complementary and alternative medicine (CAM) practitioners and self-prescribed CAM. *Clin Rheumatol* 29: 25-32.

40. Sibbritt D, Adams J, Lui CW (2011) A longitudinal analysis of complementary and alternative medicine use by a representative cohort of young Australian women with asthma, 1996-2006. *J Asthma* 48: 380-386.

41. Adams J, Sibbritt D, Broom A, Loxton D, Wardle J, et al. (2013) Complementary and alternative medicine consultations in urban and nonurban areas: a national survey of 1427 Australian women. *J Manipulative Physiol Ther* 36: 12-19.

42. Beatty LJ, Adams J, Sibbritt D, Wade TD (2012) Evaluating the impact of cancer on complementary and alternative medicine use, distress and health related QoL among Australian women: A prospective longitudinal investigation. *Complement Ther Med* 20: 61-69.

43. Broom AF, Kirby ER, Sibbritt DW, Adams J, Refshauge KM (2012) Use of complementary and alternative medicine by mid-age women with back pain: a national cross-sectional survey. *BMC Complement Altern Med* 12: 98.

44. Dunstan DW, Zimmet PZ, Welborn TA, Cameron AJ, Shaw J, et al. (2002) The Australian Diabetes, Obesity and Lifestyle Study (AusDiab)--methods and response rates. *Diabetes Res Clin Pract* 57: 119-129.

45. Briganti EM, Kerr PG, Shaw JE, Zimmet PZ, Atkins RC (2005) Prevalence and treatment of cardiovascular disease and traditional risk factors in Australian adults with renal insufficiency. *Nephrology (Carlton)* 10: 40-47.

46. Briganti EM, Shaw JE, Chadban SJ, Zimmet PZ, Welborn TA, et al. (2003) Untreated hypertension among Australian adults: the 1999-2000 Australian Diabetes, Obesity and Lifestyle Study (AusDiab). *Med J Aust* 179: 135-139.

47. Chen L, Rogers SL, Colagiuri S, Cadilhac DA, Mathew TH, et al. (2008) How do the Australian guidelines for lipid-lowering drugs perform in practice? Cardiovascular disease risk in the AusDiab Study, 1999-2000. *Med J Aust* 189: 319-322.

48. Panchapakesan J, Mitchell P, Tumuluri K, Rochtchina E, Foran S, et al. (2003) Five year incidence of cataract surgery: the Blue Mountains Eye Study. *Br J Ophthalmol* 87: 168-172.

49. Cumming RG, Mitchell P (1997) Hormone replacement therapy, reproductive factors, and cataract. The Blue Mountains Eye Study. *Am J Epidemiol* 145: 242-249.

50. Cumming RG, Mitchell P (1998) Medications and cataract. The Blue Mountains Eye Study. *Ophthalmology* 105: 1751-1758.

51. Cumming RG, Mitchell P, Leeder SR (1997) Use of inhaled corticosteroids and the risk of cataracts. *N Engl J Med* 337: 8-14.

52. Hourihan F, Mitchell P (1999) Factors associated with use of glaucoma medications in a population of older people: The Blue Mountains Eye Study. *Aust N Z J Ophthalmol* 27: 176-179.

53. Leung H, Wang JJ, Rochtchina E, Wong TY, Klein R, et al. (2004) Does hormone replacement therapy influence retinal microvascular caliber? *Microvasc Res* 67: 48-54.

54. Mitchell P, Wang JJ, Cumming RG, House P, England JD (2000) Long-term topical timolol and blood lipids: the Blue Mountains Eye Study. *J Glaucoma* 9: 174-178.

55. Smith W, Mitchell P, Wang JJ (1997) Gender, oestrogen, hormone replacement and age-related macular degeneration: results from the Blue Mountains Eye Study. *Aust N Z J Ophthalmol* 25 Suppl 1: S13-15.

56. Wu KH, Wang JJ, Rochtchina E, Foran S, Ng MK, et al. (2004) Angiotensin-converting enzyme inhibitors (ACEIs) and age-related maculopathy (ARM): cross-sectional findings from the Blue Mountains Eye Study. *Acta Ophthalmol Scand* 82: 298-303.

57. Gopinath B, Flood VM, Teber E, McMahon CM, Mitchell P (2011) Dietary Intake of Cholesterol Is Positively Associated and Use of Cholesterol-Lowering Medication Is Negatively Associated with Prevalent Age-Related Hearing Loss. *J Nutr* 141: 1355-1361.

58. Kanthan GL, Wang JJ, Rochtchina E, Mitchell P (2009) Use of antihypertensive medications and topical beta-blockers and the long-term incidence of cataract and cataract surgery. *Br J Ophthalmol* 93: 1210-1214.

59. Kanthan GL, Wang JJ, Burlutsky G, Rochtchina E, Cumming RG, et al. (2010) Exogenous oestrogen exposure, female reproductive factors and the long-term incidence of cataract: the Blue Mountains Eye Study. *Acta Opthalmologica* 88: 773-778.

60. Liew G, Mitchell P, Leeder SR, Smith W, Wong TY, et al. (2006) Regular aspirin use and retinal microvascular signs: the Blue Mountains Eye Study. *J Hypertens* 24: 1329-1335.

61. Liew G, Mitchell P, Wong TY, Rochtchina E, Wang JJ (2013) The association of aspirin use with age-related macular degeneration. *JAMA Intern Med* 173: 258-264.

62. van Leeuwen R, Tomany SC, Wang JJ, Klein R, Mitchell P, et al. (2004) Is medication use associated with the incidence of early age-related maculopathy? Pooled findings from 3 continents. *Ophthalmology* 111: 1169-1175.

63. Wang JJ, Mitchell P, Smith W, Gillies M, Billson F, et al. (2003) Systemic use of anti-inflammatory medications and age-related maculopathy: the Blue Mountains Eye Study. *Ophthalmic Epidemiol* 10: 37-48.

64. Wang JJ, Rochtchina E, Tan AG, Cumming RG, Leeder SR, et al. (2009) Use of inhaled and oral corticosteroids and the long-term risk of cataract. *Ophthalmology* 116: 652-657.

65. Younan C, Mitchell P, Cumming RG, Panchapakesan J, Rochtchina E, et al. (2002) Hormone replacement therapy, reproductive factors, and the incidence of cataract and cataract surgery: the Blue Mountains Eye Study. *Am J Epidemiol* 155: 997-1006.

66. Buyken AE, Flood V, Rochtchina E, Nestel P, Brand-Miller J, et al. (2010) Modifications in Dietary Fat Quality Are Associated with Changes in Serum Lipids of Older Adults Independently of Lipid Medication. *J Nutr* 140: 88-94.

67. Chandrasekaran S, Cumming RG, Rochtchina E, Mitchell P (2006) Associations between elevated intraocular pressure and glaucoma, use of glaucoma medications, and 5-year incident cataract: the Blue Mountains Eye Study. *Ophthalmology* 113: 417-424.

68. Tan JS, Mitchell P, Rochtchina E, Wang JJ (2007) Statin use and the long-term risk of incident cataract: the Blue Mountains Eye Study. *Am J Ophthalmol* 143: 687-689.

69. Tan JS, Mitchell P, Rochtchina E, Wang JJ (2007) Statins and the long-term risk of incident age-related macular degeneration: the Blue Mountains Eye Study. *Am J Ophthalmol* 143: 685-687.

70. Kuzniarz M, Mitchell P, Cumming RG, Flood VM (2001) Use of vitamin supplements and cataract: the Blue Mountains Eye Study. *Am J Ophthalmol* 132: 19-26.

71. Kuzniarz M, Mitchell P, Flood VM, Wang JJ (2002) Use of vitamin and zinc supplements and age-related maculopathy: the Blue Mountains Eye Study. *Ophthalmic Epidemiol* 9: 283-295.

72. Christensen H, Mackinnon A, Jorm AF, Korten A, Jacomb P, et al. (2004) The Canberra Longitudinal Study: Design, Aims, Methodology, Outcomes and Recent Empirical Investigations. *Aging Neuropsychol Cogn* 11: 169-195.

73. Henderson AS, Jorm AF, Christensen H, Jacomb PA, Korten AE (1997) Aspirin, anti-inflammatory drugs and risk of dementia. *Int J Geriatr Psychiatry* 12: 926-930.

74. Cumming RG, Handelsman D, Seibel MJ, Creasey H, Sambrook P, et al. (2009) Cohort Profile: the Concord Health and Ageing in Men Project (CHAMP). *Int J Epidemiol* 38: 374-378.

75. Bleicher K, Naganathan V, Cumming RG, Seibel MJ, Sambrook PN, et al. (2010) Prevalence and treatment of osteoporosis in older Australian men: findings from the CHAMP study. *Med J Aust* 193: 387-391.

76. Bleicher K, Cumming RG, Naganathan V, Seibel MJ, Sambrook PN, et al. (2011) Lifestyle factors, medications, and disease influence bone mineral density in older men: findings from the CHAMP study. *Osteoporos Int* 22: 2421-2437.

77. Gnjidic D, Cumming RG, Le Couteur DG, Handelsman DJ, Naganathan V, et al. (2009) Drug Burden Index and physical function in older Australian men. *Br J Clin Pharmacol* 68: 97-105.

78. Gnjidic D, Le Couteur DG, Hilmer SN, Cumming RG, Blyth FM, et al. (2014) Sedative load and functional outcomes in community-dwelling older Australian men: the CHAMP study. *Fundam Clin Pharmacol* 28: 10-19.

79. Gnjidic D, Le Couteur DG, Naganathan V, Cumming RG, Creasey H, et al. (2012) Effects of drug burden index on cognitive function in older men. *J Clin Psychopharmacol* 32: 273-277.

80. Ilomaki J, Gnjidic D, Hilmer SN, Le Couteur DG, Naganathan V, et al. (2013) Psychotropic drug use and alcohol drinking in community-dwelling older Australian men: The CHAMP study. *Drug Alcohol Rev* 32: 218-222.

81. Gnjidic D, Hilmer SN, Blyth FM, Naganathan V, Cumming RG, et al. (2012) High-risk prescribing and incidence of frailty among older community-dwelling men. *Clin Pharmacol Ther* 91: 521-528.

82. Gnjidic D, Hilmer SN, Blyth FM, Naganathan V, Waite L, et al. (2012) Polypharmacy cutoff and outcomes: five or more medicines were used to identify community-dwelling older men at risk of different adverse outcomes. *J Clin Epidemiol* 65: 989-995.

83. Gnjidic D, Le Couteur DG, Blyth FM, Travison T, Rogers K, et al. (2013) Statin use and clinical outcomes in older men: a prospective population-based study. *BMJ Open* 3: e002333. doi:10.1136/bmjopen-2012-002333

84. Gnjidic D, Stanaway FF, Cumming R, Waite L, Blyth F, et al. (2012) Mild cognitive impairment predicts institutionalization among older men: a population-based cohort study. *PLoS One* 7: e46061. doi:10.1371/journal.pone.0046061

85. Norman PE, Flicker L, Almeida OP, Hankey GJ, Hyde Z, et al. (2009) Cohort Profile: The Health In Men Study (HIMS). *Int J Epidemiol* 38: 48-52.

86. Almeida OP, Flicker L, Yeap BB, Alfonso H, McCaul K, et al. (2012) Aspirin decreases the risk of depression in older men with high plasma homocysteine. *Transl Psychiatry* 2: e151.

87. Beer C, Hyde Z, Almeida OP, Norman P, Hankey GJ, et al. (2011) Quality use of medicines and health outcomes among a cohort of community dwelling older men: an observational study. *Br J Clin Pharmacol* 71: 592-599.

88. McEvoy M, Smith W, D'Este C, Duke J, Peel R, et al. (2010) Cohort profile: The Hunter Community Study. *Int J Epidemiol* 39: 1452-1463.

89. McGettigan P, Lincz LF, Attia J, McElduff P, Bissett L, et al. (2011) The risk of coronary thrombosis with cyclo-oxygenase-2 inhibitors does not vary with polymorphisms in two regions of the cyclo-oxygenase-2 gene. *Br J Clin Pharmacol* 72: 707-714.

90. Browning CJ, Kendig H (2010) Cohort profile: the Melbourne Longitudinal Studies on Healthy Ageing Program. *Int J Epidemiol* 39: e1-7.

91. Thomson JA, Wang WC, Browning C, Kendig HL (2010) Self-reported medication side effects in an older cohort living independently in the community--the Melbourne Longitudinal Study on Healthy Ageing (MELSHA): cross-sectional analysis of prevalence and risk factors. *BMC Geriatr* 10: 37.

92. Atlantis E, Browning C, Sims J, Kendig H (2010) Diabetes incidence associated with depression and antidepressants in the Melbourne Longitudinal Studies on Healthy Ageing (MELSHA). *Int J Ger Psychiatry* 25: 688-696.

93. Atlantis E, Grayson DA, Browning C, Sims J, Kendig H (2011) Cardiovascular disease and death associated with depression and antidepressants in the Melbourne Longitudinal Studies on Healthy Ageing (MELSHA). *Int J Ger Psychiatry* 26: 341-350.

94. Slade GD, Gansky SA, Spencer AJ (1997) Two-year incidence of tooth loss among South Australians aged 60+ years. *Community Dent Oral Epidemiol* 25: 429-437.

95. Thomson WM, Chalmers JM, Spencer AJ, Slade GD (2000) Medication and dry mouth: findings from a cohort study of older people. *J Public Health Dent* 60: 12-20.

96. Thomson WM, Spencer AJ, Slade GD, Chalmers JM (2002) Is medication a risk factor for dental caries among older people? *Community Dent Oral Epidemiol* 30: 224-232.

97. Thomson WM, Chalmers JM, Spencer AJ, Slade GD, Carter KD (2006) A longitudinal study of medication exposure and xerostomia among older people. *Gerodontology* 23: 205-213.

98. Sachdev PS, Brodaty H, Reppermund S, Kochan NA, Trollor JN, et al. (2010) The Sydney Memory and Ageing Study (MAS): Methodology and baseline medical and neuropsychiatric characteristics of an elderly epidemiological non-demented cohort of Australians aged 70-90 years. *Int Psychogeriatr* 22: 1248-1264.

99. Haerer W, Delbaere K, Bartlett H, Lord SR, Rowland J (2012) Relationships between HMG-CoA reductase inhibitors (statin) use and strength, balance and falls in older people. *Intern Med J* 42: 1329-1334.

100. Wong AKW, Lord SR, Sturnieks DL, Delbaere K, Trollor JN, et al. (2013) Angiotensin System–Blocking Medications Are Associated with Fewer Falls over 12 Months in Community-Dwelling Older People. *J Am Geriatr Soc* 61: 776-781.
